# Laparoscopy in India – a personal perspective

**DOI:** 10.4103/0972-9941.16526

**Published:** 2005-06

**Authors:** Tehemton E. Udwadia

**Affiliations:** Emeritus Professor, Department of Surgery, Grant Medical College and JJ Hospital, Consultant Surgeon, Head, Department of Minimal Access Surgery, P.D. Hinduja National Hospital, Consultant Surgeon, Parsee General Hospital Breach Candy Hospital, Mumbai, India

When in 1901 Kelling[1] inserted a cystoscope into the peritoneal cavity of a dog and instilled air to examine the abdominal contents could he have visualized that before the century was over his pioneering experiment would give direction to the most significant patient-friendly revolution in the history of surgery? In the history of surgical progress there have been only three patient-friendly revolutions:

1. **Anaesthesia** which made all surgery patient-friendly.
2. **Asepsis** which saved the patient from infection, at that time the most common and deadly factor of postoperative morbidity and mortality.
3. **Laparoscopic Surgery** which by minimizing pain, medication, hospitalisation, time off from work, has elevated surgery to a new patient-friendly plane.

Less than 50 years after Kelling's experiment, Dr. F.P. Antia, then Physician at the KEM Hospital, Mumbai performed a diagnostic laparoscopy on a patient with cirrhosis using a Nitze-type telescope and a feeble filament light bulb and atmospheric air instilled with the help of a sigmoidoscope pump for induction of pneumoperitoneum. Dr. F.P. Antia compensated for the poor quality of his equipment by virtue of his legendary tenacity and perseverance, and mustered a large volume of clinical diagnostic laparoscopy both visual, and on target biopsy. Though a physician, he had realised the importance of diagnostic laparoscopy for surgeons, and published some of his results in surgical journals.[2] Unfortunately his advice to surgeons went unheeded for over 20 years.

In 1971 while waiting for the anaesthetist at the Breach Candy Hospital I saw Dr. N.D. Motashaw, the Honorary Gynaecologist at the KEM Hospital perform a diagnostic laparoscopy using a Storz laparoscope, and was amazed at the extent and clarity of vision. In 1971 when I was an Associate Honorary Surgeon at the JJ Hospital, our ward had patients on beds, between beds and in the corridors. A major contributing factor to the overcrowding was the delay in or even lack of investigative facilities. I felt that laparoscopic diagnosis as shown me by Dr. Motashaw could hasten diagnosis and treatment and ensure a more rapid patient turnover. It was with this in view that in 1972 I went to Germany and obtained the Storz laparoscopy set. Till I retired in 1993 from the JJ Hospital, this set was permanently housed in that Hospital, and used solely for the patients in the JJ Hospital. After purchasing the equipment from Storz I did not have the finances to purchase a pneumoperitoneum insufflator. Dr. F.P. Antia, who in 1972 was the Honorary Gastroenterologist at the BYL Nair Hospital invited me to attend his laparoscopy sessions and it was from him that I learnt the use of the sigmoidoscope pump to create pneumoperitoneum for diagnostic laparoscopy, a technique I used for 18 years and in well over 3000 cases.

I soon realised the value of diagnostic laparoscopy in a surgical unit in a developing country and tried to pass on my enthusiasm to all my colleagues. Surgeons in large cities viewed my passion with indifference if not scorn. To my gratification surgeons in small towns, in the course of innumerable workshops, were very receptive, specially so since they lacked other diagnostic facilities and very many of them had laparoscopy equipment as part of the family planning programme. From these somewhat primitive beginnings, laparoscopy and its logical sequel laparoscopic surgery has grown in the country in a phenomenal way. The first laparoscopic cholecystectomy in India was performed in 1990 at the JJ Hospital, Mumbai, followed a few months later in Pune by Dr. Jyotsna Kulkarni. The first workshop in minimal access surgery (MAS) in a teaching hospital was held at the KEM Hospital by Dr. J. B. Agarwal and Dr. A. Dalvi, and in a private hospital at the P. D. Hinduja National Hospital, Mumbai. Viewed at first with apprehension and scepticism by surgeons and patients alike, the gratifying results of the early cases soon converted the surgical community to ardent believers, a conversion largely propelled by patient demand.

In 1993, a small group of laparoscopic surgeons met in Mumbai to form the Indian Association of Gastrointestinal Endo-Surgeons with Dr. T. E. Udwadia as Founder President, JB Agarwal as Honorary Secretary, and Dr. H. G. Doctor as Honorary Treasurer. In one decade the membership, finances, activities and stature of this Association embraced the entire country in a brotherhood of like-minded surgeons. This Association has given tremendous thrust to laparoscopic surgery in India, has brought out a comprehensive book of Guidelines for this surgery and its members from all parts of the country have been ambassadors of Indian Laparoscopic Surgery all over the world. In terms of spread throughout the country, the volume and quality of advanced laparoscopic surgery, technical excellence, and cost-effective results, India is at the forefront of MAS-practising countries.

Our euphoria at our MAS prowess should not blind us to the realities of the wide spectrum of surgery in this country of over one billion people. Two aspects need strong emphasis:

1. Laparoscopic surgery is not a super speciality—it is merely the logical progress of general surgery brought about by advanced technology in instrumentation and imaging. To make this advance available to the entire Indian community irrespective of socio-economic status, it is imperative to spread this advance to every surgeon in India - a goal which can only be achieved if EVERY teaching hospital imparts training in MAS to every resident and EVERY University incorporates this advance as an essential element in its curriculum. As important as spreading this surgery to every surgeon is the necessity to tailor the cost of this surgery by improvisation, to try and give the benefit of this surgery to all our people. This is the real challenge faced by surgeons in India, to realize that laparoscopic surgery can grow in India not with robots but with basic equipment, to go beyond merely following the developed world by devising technology compatible with our country.
2. That a procedure can be done by laparoscopy is not adequate indication to do so. Every laparoscopic procedure must be evaluated and appraised not merely on its feasibility nor by the enthusiasm or euphoria of personal ego or achievement, but rather as a pragmatic clinical study as it applies to our own country and conditions. The history of surgery is replete with many surgical procedures practiced ardently over several decades which subsequently fell by the wayside. Time and an adequate honest follow-up alone will tell us which procedure enters the register of established and accepted practice.

Having said all this, there can be no doubt that MAS is the most compelling and dynamic force driving surgical progress and endeavour in the current era.
